# Publication, funding, and experimental data in support of Human Reference Atlas construction and usage

**DOI:** 10.1038/s41597-024-03416-8

**Published:** 2024-06-04

**Authors:** Yongxin Kong, Katy Börner

**Affiliations:** 1 Department of Intelligent Systems Engineering, Luddy School of Informatics, Computing, and Engineering, Indiana University, Bloomington, IN 47408 USA; 2 School of Information Management, Sun Yat-sen University, Guangzhou, 510006 China

**Keywords:** Data mining, Data acquisition, Cell biology

## Abstract

Experts from 18 consortia are collaborating on the Human Reference Atlas (HRA) which aims to map the 37 trillion cells in the healthy human body. Information relevant for HRA construction and usage is held by experts, published in scholarly papers, and captured in experimental data. However, these data sources use different metadata schemas and cannot be cross-searched efficiently. This paper documents the compilation of a dataset, named HRAlit, that links the 136 HRA v1.4 digital objects (31 organs with 4,279 anatomical structures, 1,210 cell types, 2,089 biomarkers) to 583,117 experts; 7,103,180 publications; 896,680 funded projects, and 1,816 experimental datasets. The resulting HRAlit has 22 tables with 20,939,937 records including 6 junction tables with 13,170,651 relationships. The HRAlit can be mined to identify leading experts, major papers, funding trends, or alignment with existing ontologies in support of systematic HRA construction and usage.

## Background & Summary

Constructing an atlas of the healthy human body is a massive undertaking due to the multiscale, biological complexity of human physiology. Since March 2020, international experts funded by the National Institutes of Health and/or supported by the Human Cell Atlas have been collaborating on the construction of a Human Reference Atlas (HRA)^1^. The 5th release of the HRA (v1.4) was published in June 2023 and comprises 31 organs with 4,279 unique anatomical structures, 1,210 unique cell types, 2,089 unique biomarkers linked to 32 Anatomical Structures, Cell Types, plus Biomarkers (ASCT + B) tables, 21 two-dimensional functional tissue units (FTU), and 65 three-dimensional, anatomically correct reference organs^2^. A total of 101 experts created and 99 experts reviewed (158 unique experts with ORCID IDs) the HRA digital objects across all releases and compiled 420 papers with DOIs that provide scholarly evidence for the anatomical structures, cell types, and biomarkers in the 31 ASCT + B tables.

As the HRA grows in the number of organs and data types it captures, it becomes important to use data-driven decision making to ensure systematic and efficient collaboration of scholars from different areas of research and development; federation of experimental data from different laboratories and data portals across scales (whole body to subcellular); and strategic foresight when setting data acquisition, tool development, and funding priorities.

In parallel to atlas construction, many high-quality experimental datasets are becoming available via data portals developed and served by Human BioMolecular Atlas Program (HuBMAP)^3^, Cellular Senescence Network (SenNet)^4^, Kidney Precision Medicine Project (KPMP)^5,6^, GenitoUrinary Developmental Molecular Anatomy Project (GUDMAP)^7^, the Genotype-Tissue Expression (GTEx)^8^, or CZ CELLxGENE^9^. However, the portals use different metadata schemas and few provide DOIs for papers and only some offer API access—searching for data across portals is difficult or impossible.

Moreover, HRA relevant data is published in scholarly papers. Each month, more than 80,000 papers are published in PubMed making it difficult to keep track of expertise, methods, data, or code. *Scientific Data* papers typically focus on ontologies^10–12^ or experimental data^13–15^ while science of science studies commonly focus on authors, their publications, and possibly the funding that supports the research^16^. The construction of a HRA benefits from interlinking expertise, publication, funding, and experimental datasets.

This paper details the construction of the HRAlit database that links the 295 digital objects of the HRA (versions 1.0 to 1.4) to publication, funding, and experimental data in support of HRA construction and usage. Linking of experimental data to scientific publications is critical in providing context and detail for how tissue data was acquired, preprocessed, what assay types were run, and how the computational analyses and visualizations can be reproduced. It makes it possible to attribute scholarly credits to experts who share experimental data and/or spend much time and energy on the generation and provisioning of HRA digital objects such as ASCT + B tables or Organ Mapping Antibody Panels (OMAPs) which are DOI’d but have no official way to accumulate citation counts.

Specifically, HRAlit includes 7,103,180 PubMed publications retrieved by a query for all 31 organs plus papers published in HRA, CZ CELLxGENE, GTEx, and CellMarker that are linked to 583,117 authors from 26,235 (cleaned) institutions and 896,680 funded projects by 6,427 (cleaned) funders. Cleaning is performed using OpenAlex^17^ (https://openalex.org) see Methods. HRAlit also links the HRA to 1,816 experimental datasets and the 4,639 healthy adult donors that volunteered their data. The anatomical structures, cell types, and biomarkers in the 5th HRA release link to 7,578 ontology IRIs (Internationalized Resource Identifier). The resulting database has 22 tables with 20,939,937 records plus 6 junction tables with 13,170,651 relationships—including relationships between publications and organs, publications and datasets, publications and authors, authors and institutions, funding and funders, among anatomical structures, cell types, and biomarkers. A data dictionary can be found in Supplemental Table S1; for record count, node count, and linkage count see Supplemental Tables S2-S4.

HRAlit is actively used to support HRA construction and usage. For example, HRAlit records what scholarly publication and experimental data evidence exists for which digital objects in the HRA; makes it possible to identify experts around the globe that might like to serve as HRA authors and reviewers—making the HRA more equitable; helps prioritize adding organs to the HRA for which sufficient experimental data and funding resources exist; guides the acquisition of new tissue data in support of HRA construction—single cell data analysis is still rather expensive and data-driven decision making does literally save millions of dollars as we can identify what data is most valuable for improving atlas quality and coverage; links HRA to existing ontologies so those can be expanded or used for ontological reasoning; and provides canonical data when exploring or annotating experimental data.

## Methods

The HRAlit database was constructed solely using publicly available metadata for publications, HRA digital objects, etc. for which human experts consented to have their names attributed.

**Fig. 1:**
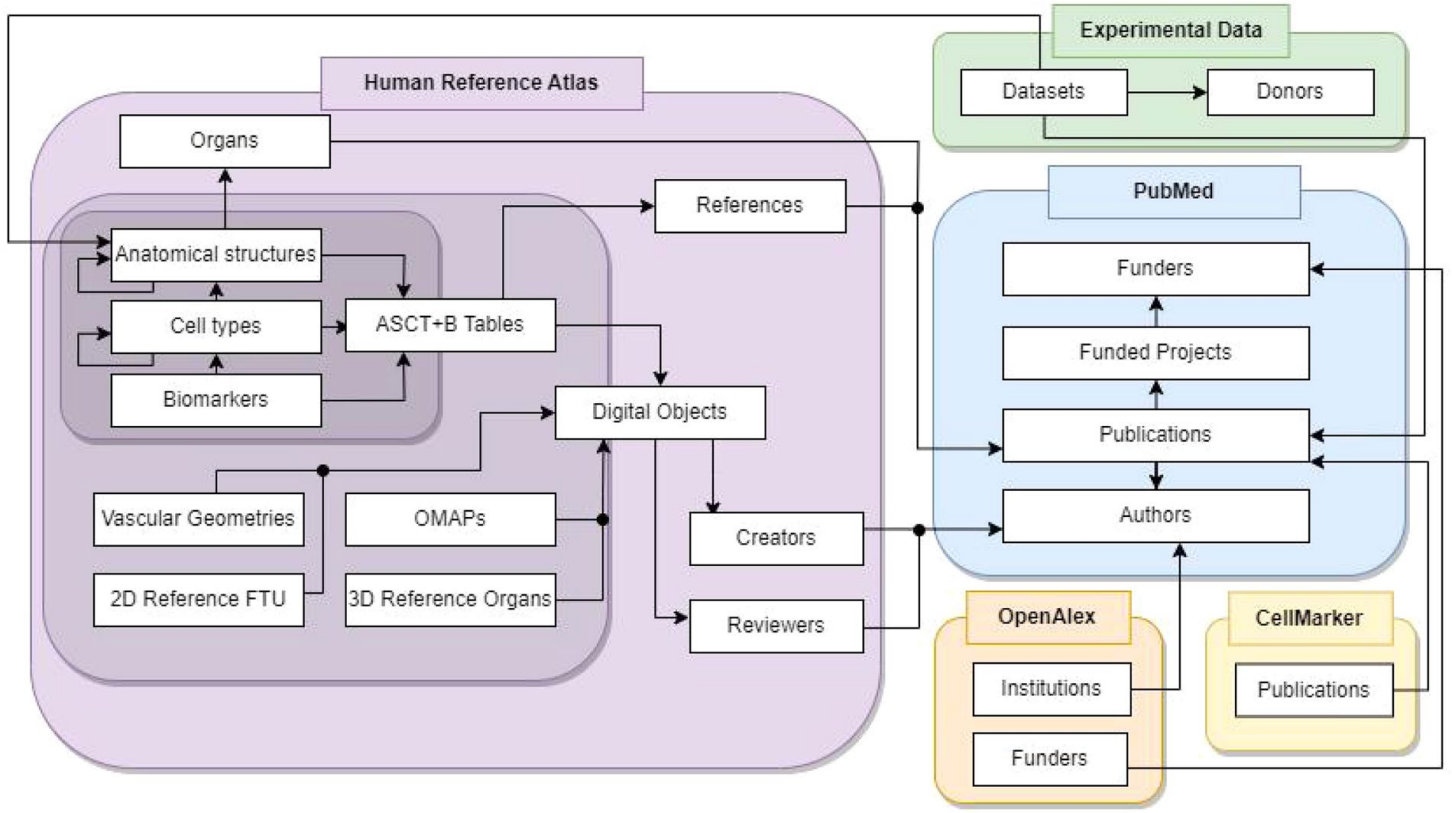
Overview of the HRAlit database. HRA data types are linked to experimental data and to PubMed data using HuBMAP IDs of the HRA digital objects; experimental dataset IDs, donor IDs, and publication DOIs; PubMed publication PMIDs, author IDs, funding IDs, institution IDs, and funder IDs.

An overview of the HRAlit database is given in Fig. 1. The database links data from the Human Reference Atlas (in left) to experimental data (top, right) and publication and funding data in PubMed; OpenAlex is used for cleaning institution and funder names; CellMarker publications are a proper subset of PubMed and they are used for technical validation.

**Fig. 2:**
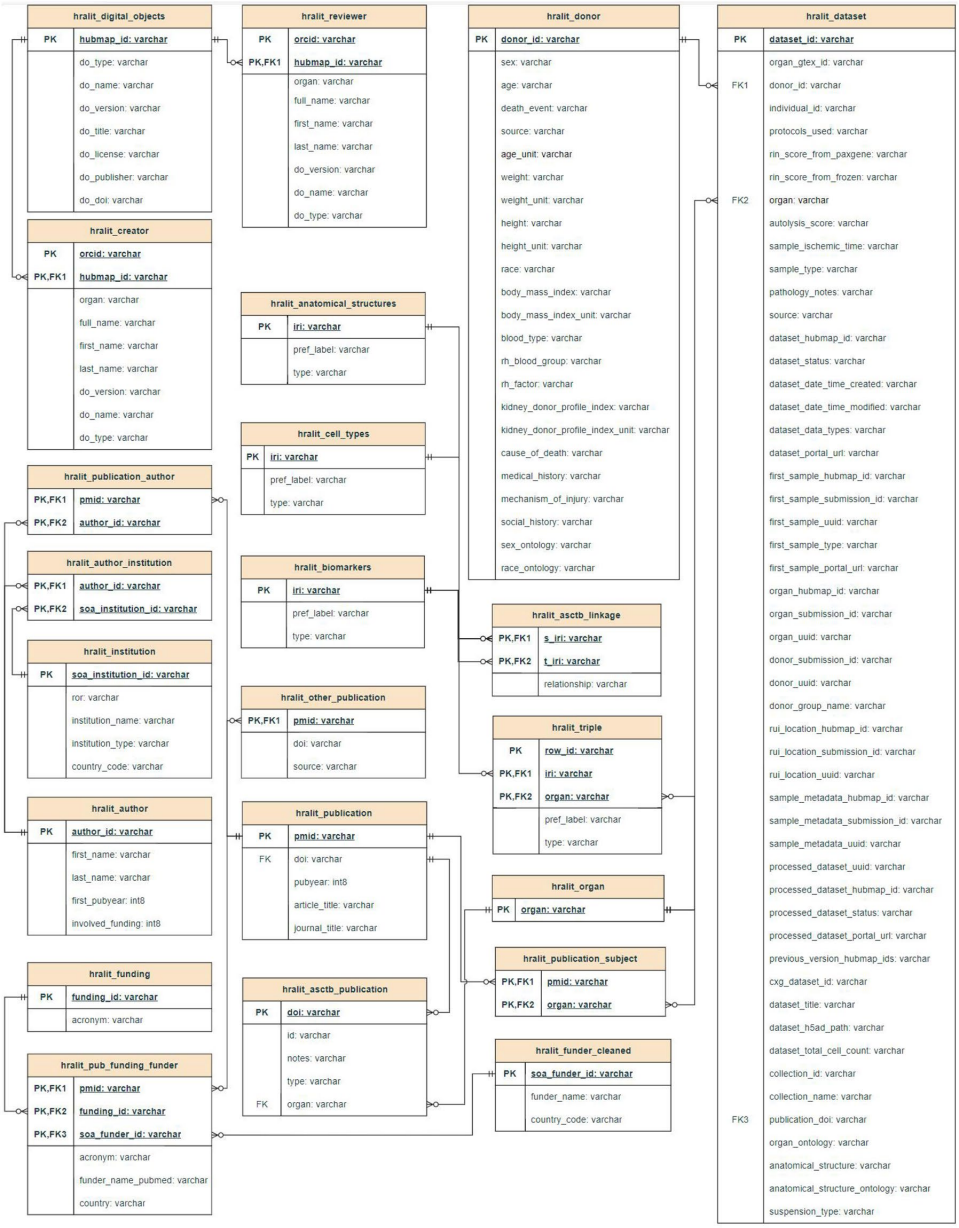
Entity relationship diagram of the HRAlit database. Shown are the 22 entity tables including 6 junction tables and their properties.

The entity relationship diagram—a graphical representation of the entity types and their relationships—of the HRAlit database is given in Fig. 2. There are 22 tables, each with a name (e.g., “hralit_digital_objects”), primary keys (PK, e.g., “hubmap_id”) used to uniquely identify each record, foreign keys (FK, e.g., “pmid”) used to reference the primary key of another table, and all entity properties (e.g., “do_type”). Six of these tables are so-called junction tables that capture relationships (e.g., “hralit_publication_author” table links “pmid” and “author_id”).

For HRA experts such as “hralit_creator” and “hralit_reviewer” tables, there exist “orcid” identifiers; for all other tables an “author_id” is used to refer to experts.

Subsequently, we detail data download and preprocessing, database construction, and database usage.

### Data download and preprocessing

We detail here the step-by-step addition of all data types and associated linkages.

#### Human reference atlas

**HRA digital objects** from the HRA 5th release (v1.4) were downloaded from https://hubmapconsortium.github.io/ccf-releases/v1.4/docs and include 32 ASCT + B Tables (including 1 crosswalk table), 21 Functional Tissue Units (FTUs), 13 Organ Mapping Antibody Panels (OMAPs), 1 Vascular Geometries table, as well as 65 3D Reference Organs plus 3 digital objects for two united and 1 crosswalk from the 3D organs to the ASCT + B tables for a total of 136. All 295 HRA digital objects across all HRA releases (starting with v1.0 in March 2021) can be accessed via CCF-HRA releases^18^ and extracted as a HRA metadata table.

**HRA Experts**. Information about the creators and reviewers of the HRA digital objects (e.g., ORCIDs, first names, and last names, associated digital objects) across all HRA versions was parsed from the metadata table that covers all HRA digital objects. In total, there are 101 unique creators and 99 unique reviewers for a total of 158 unique experts.

**HRA Publications**. In the HRA, publication references are classified as “general reference” that supports organ-specific ASCT + B tables and are listed in the table header and “specific reference” papers that support combinations of table row-specific anatomical structures, cell types, and biomarkers triples. In total, the 5th release ASCT + B tables have 83 general references and 1,070 specific references for a total of 1,057 unique publication references, see https://ccf-ontology.hubmapconsortium.org/v2.2.1/ccf-asctb-all.json. Some of these publications are books (e.g., anatomy books) and Wikipedia entries if no other sources exist. Exactly 420 of the publications have DOIs and 391 are in PubMed. Each publication is stored with an ID, DOI, accompanying notes, categorization type, and an associated organ.

**HRA Ontologies**. Anatomical structures, cell types, and biomarkers in the 5th release HRA are linked to their counterparts in existing ontologies; for 2,619 terms there do not yet exist entries in ontologies and HRA-TEMP IDs are assigned to facilitate ID-based mapping. Issue requests exist to add these 2,619 terms to existing ontologies over the coming 1-2 years to properly represent healthy human data. Ontology IDs were extracted from data source in the 5th HRA release (https://purl.humanatlas.io/graph/ccf/v2.2.1) via LDSPARQL (https://cns-iu.github.io/ldsparql). In total, 1,277 anatomical structures are linked to the Foundational Model of Anatomy (FMA) and 1,533 to the Uber-anatomy ontology (Uberon), 646 cell types are linked to entries in the Cell Ontology (CL) and 127 Provisional Cell Ontology (PCL), 2,092 biomarkers are linked to the Human Genome Nomenclature Committee (HGNC). Additionally, four relationship types are obtained via LDSPARQL: 4,795 “part_of” relationships between 4,278 unique anatomical structures, 13,471 “located_in” relationships between 1,210 unique cell types and these anatomical structures, 1,258 “is_a” relationships between cell types, and 5,753 “characterizes” relationships between 2,076 unique biomarkers and the unique cell types.

#### CellMarker

For use in the technical validation, additional publications were downloaded from CellMarker 1.0^19^, a manually curated dataset of biomarkers for distinguishing different cell types in different anatomical structures in human and mouse derived from and linked to over 10,000 publication references. CellMarker data for human, including 1,764 PubMed IDs, was downloaded as a file from the CellMarker portal at http://bio-bigdata.hrbmu.edu.cn/CellMarker1.0.

#### Publications

PubMed (https://pubmed.ncbi.nlm.nih.gov) provides free access to publications encompassing various health and life sciences disciplines. Daily updates of PubMed can be downloaded via API (https://www.ncbi.nlm.nih.gov/books/NBK25497/). We queried a local, daily updated database on September 12, 2023 using the 31 organ names covered in the 5th release HRA as query keywords. A total of 7,101,948 publications with any of the 31 organ names in the paper title or PubMed keywords (i.e., MeSH terms) were retrieved, covering years from 1900 to 2023. In addition, we added publications from HRA, CZ CELLxGENE, GTEx, and CellMarker, see details in Database Construction section.

#### Experts

Author details were extracted from the PubMed publications using unique identifiers for authors. We only select the authors with unique identifiers, and include for each author the ID, first name, last name, earliest publication year, and number of funded projects. Specifically, the earliest publication year refers to the year of an author’s first publication indexed in PubMed; the number of funded projects represents the count of unique funded projects associated with an author’s PubMed publications. A total of 583,059 experts were identified from the 483,998 publications. Plus, authors associated with publications from HRA, CZ CELLxGENE, GTEx, CellMarker were compiled and merged, see details in Database Construction section. HRAlit internal author IDs were assigned to all PubMed authors.

#### Institutions

Author institutions provided by PubMed were cleaned using data from the OpenAlex database^19^ retrieved on August 31, 2023. OpenAlex sources data about institutions from PubMed, Crossref (https://www.crossref.org), Research Organization Registry (ROR, https://ror.org), Microsoft Academic Graph (MAG, https://makg.org), and various publisher websites, focusing on indexing institutions associated with authors. Accessible via the OpenAlex API (https://docs.openalex.org), each institution is uniquely identified using both an internal ID (e.g., https://openalex.org/I2802101240) and a canonical external ID, called ROR ID. OpenAlex provides the institution name, institution type, and country code for each registered entity. We obtained 102,494 institution entities and their associated authors’ IDs.

#### Funding and funders

PubMed also provides information on funding and funders, as well as the relationships among publications, funded projects, and their funders. We extract these linkages, which include details such as funding IDs, funding acronyms, funder countries, and funder names. For further processing, funder data was cleaned utilizing the OpenAlex database. Funder data in OpenAlex originates from Crossref, linked to funding IDs and PMIDs. OpenAlex provides an internal ID (e.g., https://openalex.org/F4320309241), funder name, and country code. We obtained 14,327 funder entities connected to 132,137 funded projects from PubMed from OpenAlex on August 31, 2023.

#### Experimental data

Tissue dataset metadata was downloaded from three public data portals on August 7, 2023.

**HuBMAP Portal** (https://portal.hubmapconsortium.org): The HuBMAP SmartAPI was used to download 1,610 open access tissue samples from 193 donors with 1,445 associated datasets using https://entity.api.hubmapconsortium.org/datasets/prov-info.

**CZ CELLxGENE Portal** (https://cellxgene.cziscience.com): Using the cellxgene-census API at https://chanzuckerberg.github.io/cellxgene-census/index.html, 593 datasets from 5,328 donors, linked to 58 publications with DOIs (52 have PubMed IDs), were downloaded.

**GTEx Portal** (https://gtexportal.org): Single cell data for 25 samples from 16 donors was downloaded manually from https://www.science.org/doi/suppl/10.1126/science.abl4290/suppl_file/science.abl4290_tables_s1_to_s20.zip^20^.

All experimental datasets used to construct HRAlit are publicly available, no member login is required to access this data.

### Database construction

Using the downloaded and preprocessed data, the HRAlit database was constructed using the following steps:

From the HRA data across five releases, including digital objects and experts, we build “hralit_digital_objects” table with 295 HRA digital object records using HuBMAP IDs as the primary key. We also construct the “hralit_creator” table with 550 HRA creator records and the “hralit_reviewer” table with 602 HRA reviewer records, including ORCIDs and associated HuBMAP IDs. We use the 31 organ names in the most recent, 5th release HRA, to construct the “hralit_organ” table. The associated “hralit_anatomical_structures”, “hralit_cell_types”, and “hralit_biomarkers” tables, use IRIs as primary key and contain 4,378 anatomical structure records, 1,395 cell type records, and 2,522 biomarker records, respectively. Each record features IRI, name, and type. We also compile the “hralit_asctb_publication” table with 1,288 records, including both general and specific references. Besides, the binary relationships “hralit_asctb_linkage” table contains four types of relationships in a total of 25,277 records, which include source IRI, relationship, and target IRI.

Subsequently, we extract the triple linkages among anatomical structures (AS), cell types (CT), and biomarkers (B) from the 5th release ASCT + B Tables. For anatomical structures, cell types, and biomarkers listed in the same row of a ASCT + B Table, we assign a unique identifier called “row_id” to each row, typically format as a prefixed namespace followed by a numerical code (e.g., “blood_11”). This information can be found in the “hralit_triple” table.

For open access experimental data from HuBMAP, CxG, and GTEx, we integrate donor metadata into the “hralit_donor” table, which contains 4,639 donor records with details such as sex and age, and uses donor ID as the primary key. Additionally, we compile dataset metadata into the “hralit_dataset” table with 7,337 records, which includes dataset ID, title, associated organ, source, among others, and provides links to donors via donor IDs and to publication via reference DOIs.

Publication data involves those retrieved from PubMed using a literature search with 31 organ names, those associated with HRA or experimental datasets, as well as those listed in CellMarker for human cell makers. We record the literature searching results in the “hralit_publication_subject” tables with 7,898,258 records of linkage between PMIDs and organs. Additionally, we construct the “hralit_other_publication” table with 1,823 records of publication in experimental datasets or CellMarker, including PMIDs, DOIs, and source. To enhance the quality of this table, we populated the null PMID and DOI fields by matching the recordings in PubMed. Subsequently, we build the “hralit_publication” to record the information on publications from “hralit_publication_subject”, “hralit_other_publication”, and “hralit_asctb_publication” tables. With our primary emphasis on PubMed publications, we exclude any publications not indexed in PubMed. This table comprises 7,103,180 records of PubMed publications, utilizing PMID as the primary key and encompassing identifiers (PMIDs and DOIs), article titles, journal titles, and publication year.

To aggregate author data, we create the “hralit_publication_author” table to associate publications from the “hralit_publication” table with authors. There are 583,059 authors with ORCID identifiers that authored 483,998 of the 7,103,180 publications.

We develop the author metadata table “hralit_author”, which aggregates authors from the PubMed associated with the publications from the “hralit_publication” table and integrates experts listed in “hralit_creator” and “hralit_reviewer” tables. In support of efficient indexing, we assign a unique ID to each author. Specifically, we compile all ORCIDs and assign each of them a running number, starting from 1. This assigned number is referred to as the HRAlit internal author ID. The “hralit_author” table records information for 583,117 authors, using “author_id” as the primary key, detailing author IDs, first names, last names, earliest publication years, the number of involved funding. Furthermore, we create the “hralit_publication_author” table to associate publications from the “hralit_publication” table with authors. There are 583,059 authors that authored 483,998 of the 7,103,180 publications. This table contains 1,079,698 records of linkage between PMIDs and author IDs.

When compiling data on institutional affiliations, we correlate authorship with respective institutions using OpenAlex data, employing authors’ identifiers (ORCID and HRAlit-Author-IDs) to ensure accurate associations. The results are available in the “hralit_author_institution” table, which contains 464,043 linkages between author IDs and OpenAlex institution IDs. Next, we build the institution metadata table “hralit_institution” to record information from OpenAlex for institutions listed in the “hralit_author_institution” table. The resulting table uses OpenAlex institution ID as the primary key and contains records for 6,235 institutions; for each institution, we list ROR ID, institution name, type of institution, and country code. The 6,235 institutions are linked to 463,617 unique author IDs covering 79.50% of 583,117 unique author IDs.

Finally, to link publications to funded projects and funders, we use the linkages between publications, funding IDs, and funders sourced from PubMed, to matching PMIDs found in the “hralit_publication” table. The resulting data can be accessed in the “hralit_pub_funding_funder” table, which is linked to 899,796 publications. Funding details are covered in “hralit_funding” that lists the 917,061 funded projects, including funding IDs and acronyms. Using cleaned funder data from OpenAlex, we add OpenAlex funder IDs to the “hralit_pub_funding_funder” table through matching the corresponding PMID and funding ID. Next, we compiled the “hralit_funder_cleaned” table for all OpenAlex funder IDs listed in the “hralit_pub_funding_funder” table. The “hralit_funder_cleaned” table utilizes OpenAlex funder IDs as its primary key, comprises 6,427 funder records and includes funder names and their country codes.

## Data Records

The HRAlit database SQL file and all tables in CSV format are at Figshare^21^.

HRAlit database has 16 entity tables (see Table 1) that are interlinked via six linkage (also called junction) tables which interrelate anatomical structures, cell types and biomarkers in the HRA via the “hralit_asctb_linkage” table with 25,277 records and the “hralit_triple” table with 178,893 records; organs in the “hralit_publication_subject” table with 7,898,258 records; associated publication data in the “hralit_publication_author” table with 1,079,698 records and “hralit_pub_funding_funder” table with 2,632,888 records; associated institution information in the “hralit_author_institution” table with 464,043 records. In total, the HRAlit database has 22 tables, see detailed descriptions of table contents and counts in Supplemental Table S1.

**Table 1:** HRAlit data types, names, number of records, and source data for 16 data tables.

Data Type	Data Table Name	#Records	Source Data*
HRA	*hralit_digital_objects*	295	Human Reference Atlas (HRA) (versions 1.0 to 1.4)
	*hralit_anatomical_structures*	4,378	Human Reference Atlas (HRA) (version 1.4)
	*hralit_cell_types*	1,395	
	*hralit_biomarkers*	2,522	
	*hralit_organ*	31	
Publication**	*hralit_publication*	7,103,180	PubMed
	*hralit_other_publication*	1,823	CellMarker, CxG, GTEx
	*hralit_asctb_publication*	1,288	5th release ASCT + B Tables v1.4
Author	*hralit_author*	583,117	PubMed
	*hralit_creator*	550	HRA (versions 1.0 to 1.4)
	*hralit_reviewer*	602	HRA (versions 1.0 to 1.4)
Institution	*hralit_institution*	26,235	PubMed, cleaned using OpenAlex
Funded Projects	*hralit_funding*	917,061	PubMed
Funder	*hralit_funder_cleaned*	6,427	PubMed, cleaned using OpenAlex
Experimental Data	*hralit_dataset*	7,337	HuBMAP, CxG, GTEx
	*hralit_donor*	4,639	
Total***	8,660,880 records		

Note that * Sources include the Human BioMolecular Atlas Program (HuBMAP), the Human Reference Atlas (HRA), CZ CELLxGENE (CxG), the Genotype-Tissue Expression (GTEx), and Anatomical Structures, Cell Types, plus Biomarkers tables (ASCT + B tables). ** PubMed Publications in the “hralit_other_publication” and “hralit_asctb_publication” tables are included in the publication metadata table (“hralit_publication” table). *** In the HRAlit database, there are 7,160,702 non-unique records in metadata tables and 12,279,057 non-unique records in junction tables for a total of 22 tables with 20,939,937 records.

HRAlit database is offered as an SQL file for users to download and use on their local SQL server environment. For each table, a CSV file is provided, enabling users to easily access and analyze data using common tools. The combination of SQL and CSV files allows for flexible data handling and is particularly useful for research applications.

A link between data fields in different tables is made when they share an identical ID (see Fig. 2). For example an author name in the “hralit_creator” table is linked to a HRA digital object in the “hralit_digital_objects table” via a shared “hubmap_id”.

## Technical Validation

In the construction of this database, multiple data sources, recognized for their reliability in biological and scholarly research, are employed. The literature is sourced from PubMed^22–24^, a database extensively used in the biomedical field. Experimental data were obtained from platforms such as CZ CELLxGENE^25–27^, HuBMAP^28,29^, and GTEx^8,30^, which provide high quality data used in HRA construction. Additionally, accurate institutional and funder information is sourced from OpenAlex^31,32^ that uses Microsoft Academic Graph (MAG)^33^ and Crossref^34^ data.

HRAlit focuses on the 31 organs and associated PubMed indexed papers in the HRA v1.4. To validate publication data, we compare it to similar commonly used datasets, such as Web of Science and OpenAlex^31,35^. WoS XML raw data for years 1898 to 2023 was acquired by the Indiana University Network Institute from Clarivate Analytics under a Data Custodian user agreement with the Collaborative Archive & Data Research Environment (CADRE)^36^. Based on the linkages between WoS IDs and PMIDs provided by WoS, citations for publications in HRAlit were computed as the total number of papers that cite a publication. Table 2 shows the coverage of the HRAlit publications in WoS and OpenAlex. Exactly 65.76% of HRAlit publications are indexed in WoS. Exactly 59.23% of the 35,444,984 publications in all of PubMed are indexed in WoS, which is due to the fact that PubMed mainly covers the biomedical sciences. The OpenAlex database uses publications from the Microsoft Academic Graph (MAG) and 56.29% of HRAlit publications are indexed, closely mirroring the 56.28% for the entire PubMed dataset.

**Table 2:** Coverage of HRAlit publications in WoS and OpenAlex databases.

Source	HRAlit	Percentage	PubMed	Percentage
PubMed	7,103,180	100%	35,444,984	100%
WoS	4,672,181	65.76%	20,993,269	59.23%
OpenAlex	3,998,441	56.29%	19,949,767	56.28%

**Fig. 3:**
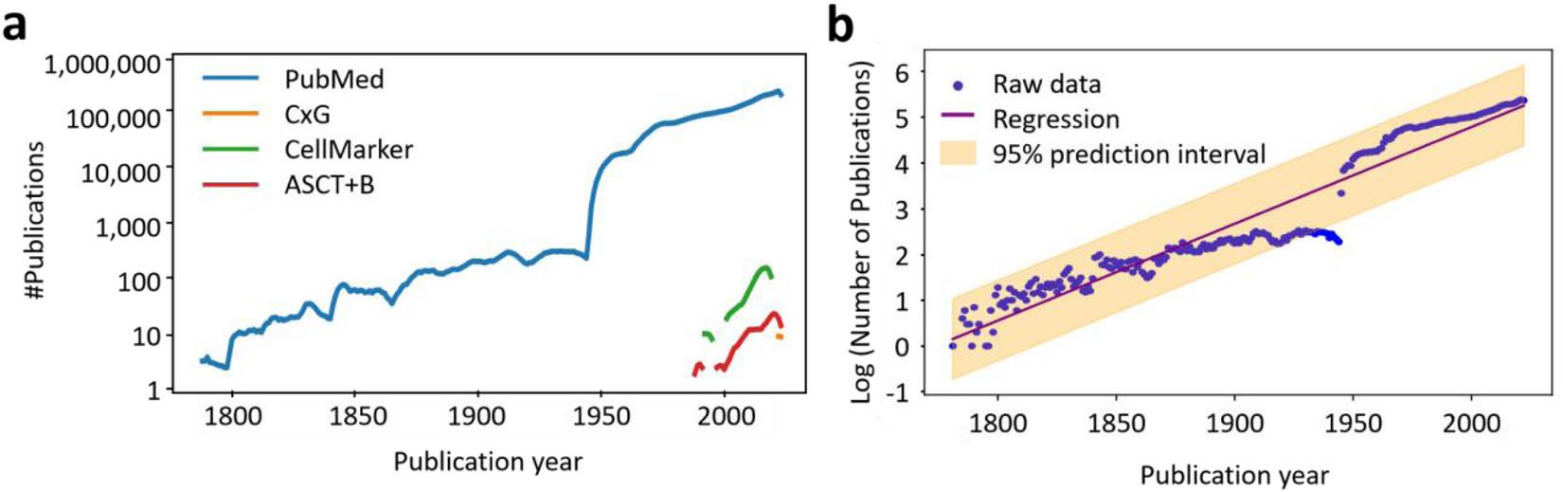
Number and growth of publications per publication year. (**a**). Number of papers published per year for the 31 organs for the four data sources. GTEx single cell data was published in a 2022 paper. (**b**). Growth in the number of publications in the HRAlit database over time.

Figure 3a shows that while PubMed publications span a broad range of periods, publications from both CellMarker and ASCT + B Tables are concentrated in recent decades, with CxG publications starting in 2018. The number of publications per year increases exponentially, consistent with results reported in prior work^37^. Figure 3b represents the results of a linear regression analysis for the growth in the number of HRAlit publications plotted on a log scale with 95% prediction interval. The annual growth rate is 4.99%, resulting in a doubling time of 14.23 years, which is typical in the Life Sciences^38^.

As the HRAlit database relies on the linkages from publications to other data, including authors, institutions, funding, and funders, we validate these linkages by comparing the HRAlit database with WoS data and OpenAlex data, see Table 3. The second column shows the number of links between PMIDs and funding IDs in the HRAlit database. The third column lists the number of links between PMIDs, funding IDs, and funder names. Exactly 78.60% of the linkages between HRAlit publications and funding in “hralit_pub_funding_funder” can also be found in WoS. For the triple relationship publications to funding to funders, there are 69.05% covered in WoS.

**Table 3:** Comparison of linkages in HRAlit and WoS databases.

	Publication - Funding	Publication - Funding - Funder	Publication - Author	Author - Institution
HRAlit	2,616,446	2,624,564	82,210*	463,617
WoS	3,328,899	3,800,778	82,209**	583,111
Percentage	78.60%	69.05%	99.99%	79.51%

Note that * Number of PMIDs that are associated with authors. ** Number of WoS publications where PMIDs are linked to an equal or larger number of authors compared to those linked to authors.

Exactly 82,210 publications with PMIDs in HRAlit are associated with 583,117 authors. The number of authors for each HRAlit publication either matched or is less than the number of authors for the equivalent WoS publication, given that WoS does not collect identifiers for authors. One exception was identified for the paper with PMID: 28842392, where the WoS database appeared to have inaccuracies—the list of names does not match the list of names on the publication with DOI:10.2196/mhealth.7254. Furthermore, 463,617 of the 583,117 authors were linked to a cleaned institution identifier sourced from OpenAlex, which is 79.51% of the total authors associated with institutions from WoS.

**Fig. 4:**
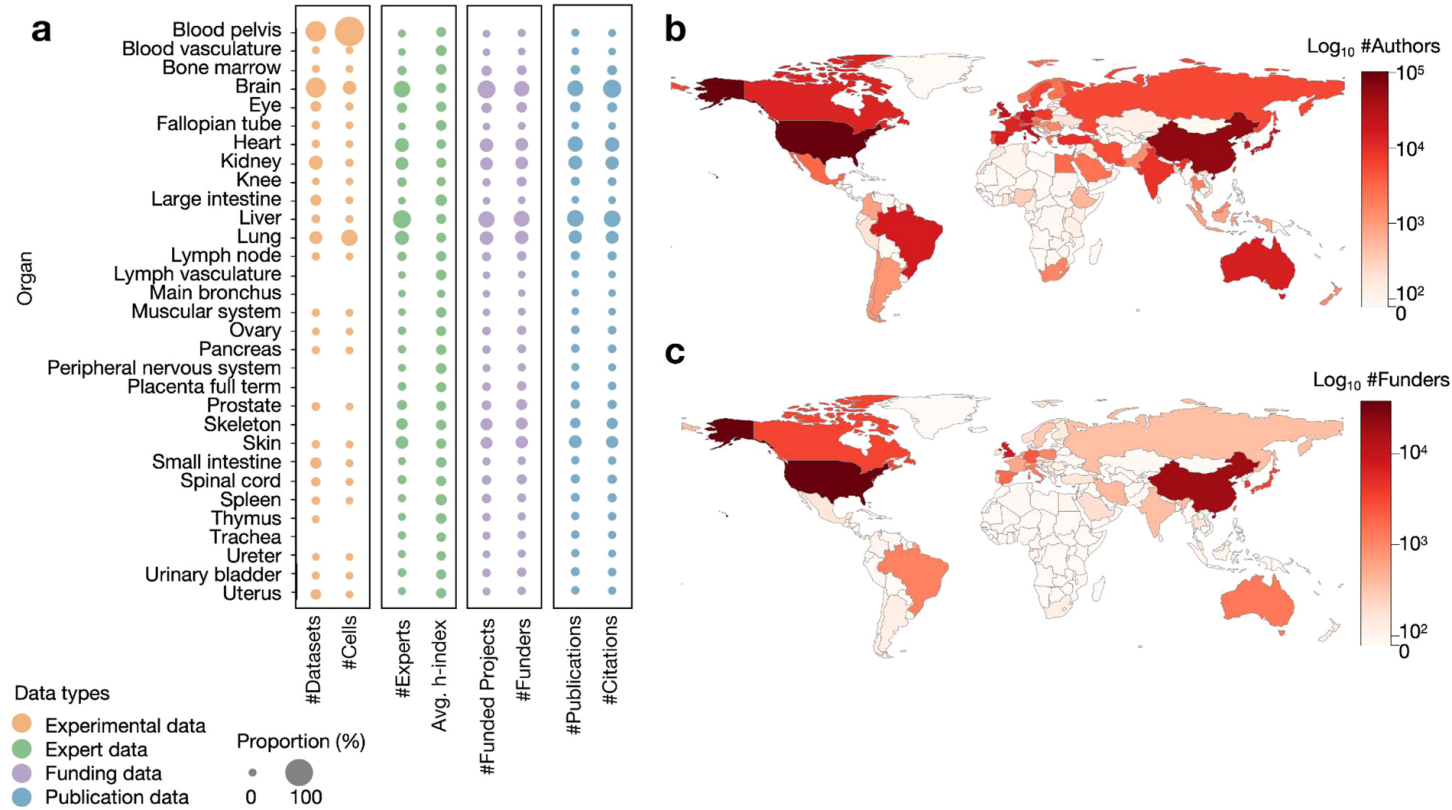
Distribution of different data types across 31 organs and choropleth map showing the number of authors and funders per country. (**a**). Experimental data, experts, funding, and publications for each of the 31 organs in the HRA. Dataset count equals the number of experimental datasets per organ in the “hralit_dataset” table. Cell count refers to the total number of cells per organ for which single cell data exists. Expert count denotes the number of experts (authors and HRA experts) (in the hralit_author” table). Funding count refers to the number of funded projects per organ that are cited by PubMed publications (in the “hralit_funding” table). Funder count equals the number of funding agencies per organ that support the funding (in the “hralit_funder_cleaned” table). Publication count equals the total count of all publications in the “hralit_publication” table. Citation count and *h*-index count were retrieved from the Web of Science core collection. (**b**). Choropleth map showing the number of paper authors overlaid on a world map. (**c**). Choropleth map for funders.

Last but not least, we validate the HRAlit database using the 31 organs covered in HRA v1.4. Figure 4a lists all 31 organs and shows counts for different data types using proportional symbols. For instance, human blood (with 3D reference organ pelvis) has the most experimental datasets. Brain, known as the most complex organ^39^, attracts much funding by different funders, which supports many experts that produce a substantial number of papers^40^. In terms of the geographical distribution of authors and funders, we see a typical distribution of scientific activity^41^ with a higher number of experts, funders, and papers in developed countries and urban centers (see Fig. 4b,c).

## Usage Notes

The HRAlit database was optimized for use in HRA construction and usage. The HRAlit detailed here was compiled using HRA v1.4 and data downloaded between August 7, 2023 and September 12, 2023. The HRAlit database will be updated as new HRA releases become available.

HRAlit publications are sourced from HRA, PubMed, CellMarker, CxG, and GTEx. In the “*hralit_publication*” table, only publications indexed in PubMed are listed. The “*hralit_asctb_publication*” table covers all references from HRA, while the “*hralit_other_publication*” table includes references from both CellMarker and CxG.

Author data is restricted to experts who have identifiers in PubMed. This decision enhances the accuracy of authorship attribution but excludes authors who do not have an identifier.

Institution and funder names were cleaned using data from OpenAlex. Although OpenAlex gathers its information from multiple datasets, including Crossref, it does not have full coverage. Cleaning funder data is particularly challenging and the choice between using cleaned or original funder names (both are provided) should be based on use case requirements.

We provide data in both SQL and CSV formats to accommodate a range of user skills, preferences, and requirements. The SQL file is recommended for complex data manipulation using database functionality while the CSV files are more appropriate for simple data access and basic data analysis.

The HRAlit can be mined to identify leading experts as potential authors of future HRA digital objects or reviewers of existing objects. It has been utilized to identify which major papers and experimental datasets exist for individual organs. Funding for different organs can be used as a proxy for predicting what experimental datasets might become available in the near future. In sum, the HRAlit supports data-driven decision making in support of systematic HRA construction and usage. While some of this data (e.g., HRA digital objects or PubMed papers) can be accessed individually, it is the global interlinkage of relevant data types that provides much additional value. Analogous to citation indexes for scholarly publications, the HRAlit database provides a citation and attribution index for the evolving HRA.

### Supplementary information

Supplementary information for Publication, Funding, and Experimental Data in Support of Human Reference Atlas Construction and Usage

## Data Availability

All code for data extraction, database construction, usage, and validation is available at https://github.com/cns-iu/hra-literature. Code is licensed under the MIT License and data under the Creative Commons Attribution 4.0 International License.
