# Supplementary material for: Publication, funding, and experimental data in support of Human Reference Atlas construction and usage
Source: Sci Data. 2024 Jun 4;11:574. doi: 10.1038/s41597-024-03416-8 (PMC11150433; doi:10.1038/s41597-024-03416-8)
Supplement: Supplementary file 1 — Supplementary information for Publication, Funding, and Experimental Data in Support of Human Reference Atlas Construction and Usage [file 41597_2024_3416_MOESM1_ESM.pdf]

Supplementary Tables

Publication, Funding, and Experimental Data in Support of Human Reference Atlas Construction and Usage

Yongxin Kong[1,2,*] and Katy Börner[1,*]

[1] Department of Intelligent Systems Engineering, Luddy School of Informatics, Computing, and Engineering, Indiana University, Bloomington, IN 47408, USA

[2] School of Information Management, Sun Yat-sen University, Guangzhou 510006, China

* Corresponding authors
Yongxin Kong, yokong@iu.edu
Katy Börner, katy@iu.edu

Experts from 18 consortia are collaborating on the Human Reference Atlas (HRA) which aims to map the 37 trillion cells in the healthy human body. Information relevant for HRA construction and usage is held by experts, published in scholarly papers, and captured in experimental data. However, these data sources use different metadata schemes and cannot be cross-searched efficiently. This paper documents the compilation of a dataset, called HRAlit, that links the 136 HRA v1.4 digital objects (31 organs with 4,279 anatomical structures, 1,210 cell types, 2,089 biomarkers) to 583,117 experts; 7,103,180 publications; 896,680 funded projects, and 1,816 experimental datasets. The resulting HRAlit has 22 tables with 20,939,937 records including 6 junction tables with 13,170,651 relationships. The HRAlit can be mined to identify leading experts, major papers, funding trends, or alignment with existing ontologies in support of systematic HRA construction and usage. Data and code are at https://figshare.com/articles/dataset/24580669 and https://github.com/cns-iu/hra-literature.

Table S1. Table description.

Table Name	Attribute	Description		
hralit_author	Purpose	To store information about authors who have contributed to publications, as well as creators and reviewers involved in the Human Reference Atlas (HRA) effort.		
	Fields	Column	Data type	Description
		author_id	varchar	Unique identifier for each author.
		first_name	varchar	Author's first name.
		last_name	varchar	Author's last name.
		first_pubyear	int8	The year in which the author's first publication appeared.
		involved_funding	int8	An integer indicating the number of funding opportunities the author has been involved in.
	Relationships	Linked to "*hralit_publication_author*" table via "*author_id*".<br>Linked to "*hralit_author_institution*" table via "*author_id*".		
	Notes	The "*involved_funding*" field indicates the number of funded projects that have supported the author's publications as of August 2023. This field will need to be updated as the author secures new funding in the future.		
hralit_publication	Purpose	To store detailed information about individual publications.		
	Fields	Column	Data type	Description
		pmid	varchar	Unique identifier for each publication based on the PubMed ID.
		doi	varchar	Digital Object Identifier for the publication.
		pubyear	int8	The year in which the publication appeared.

		article_title	varchar	Title of the article.
		journal_title	varchar	Journal in which the article was published.
	Relationships	Linked to "*hralit_publication_author*" table via "*pmid*".		
		Linked to "*hralit_publication_subject*" table via "*pmid*".		
		Linked to "*hralit_pub_funding_funder*" via "*pmid*".		
		Linked to "*hralit_asctb_publication*" via "*doi*".		
	Notes	The "*pmid*" field is a unique identifier based on the PubMed system, commonly used in biomedical literature.		
		The "*doi*" field is another unique identifier that provides a permanent link to the article.		
hralit_institution	Purpose	To store detailed information about research institutions.		
	Fields	Column	Data type	Description
		soa_institution_id	varchar	Unique identifier for each institution, based on SemOpenAlex dataset.
		ror	varchar	Research Organization Registry (ROR) identifier, a globally unique identifier for research organizations.
		institution_name	varchar	Name of the institution.
		institution_type	varchar	Type of institution (e.g., education, government, company, etc.)
		country_code	varchar	ISO country code indicating the country where the institution is located.
	Relationships	Linked to "*hralit_author_institution*" table via "*soa_institution_id*".		
	Notes	The SemOpenAlex portal is available at https://semopenalex.org.		
		The Research Organization Registry (ROR) portal can be found at https://ror.org.		
hralit_author_institution	Purpose	To establish relationships between authors and the institutions with which they are affiliated.		
	Fields	Column	Data type	Description
		author_id	varchar	Unique identifier for each author.
		soa_institution_id	varchar	Unique identifier for each institution, based on SemOpenAlex dataset.
	Relationships	Linked to "*hralit_author*" table via "*author_id*".		
		Linked to "*hralit_institution*" table via "*soa_institution_id*".		
	Notes	The Research Organization Registry (ROR) portal can be found at https://ror.org.		
hralit_funding	Purpose	To store information about various funding sources that support research.		
	Fields	Column	Data type	Description
		funding_id	varchar	Unique identifier for each funding source.
		acronym	varchar	Acronym or short name for the funding source.
	Relationships	Linked to "*hralit_pub_funding_funder*" table via "*funding_id*".		
	Notes	The "*acronym*" field can be used for easier identification and referencing of funding sources.		
hralit_funder_cleaned	Purpose	To store cleaned and standardized information about entities that fund research.		
	Fields	Column	Data type	Description
		soa_funder_id	varchar	Unique identifier for each funder, based on SemOpenAlex dataset.
		funder_name	varchar	Full name of the funder.
		country_code	varchar	ISO country code indicating the country where the funder is based.
	Relationships	Linked to "*hralit_pub_funding_funder*" table via "*soa_funder_id*".		
	Notes	The data for this table comes from SemOpenAlex.		
hralit_pub_fund	Purpose	To link publications with their respective funding sources and funders.		

ing_funder	Fields	Column	Data type	Description
		pmid	varchar	Unique identifier for each publication based on the PubMed ID.
		funding_id	varchar	Unique identifier for each funding source.
		acronym	varchar	Acronym or short name for the funding source.
		funder_name_pubmed	varchar	Name of the funder as it appears in PubMed.
		soa_funder_id	varchar	Unique identifier for each funder, based on SemOpenAlex dataset.
		country	varchar	Country where the funder is based.
	Relationships	Linked to "*hralit_publication*" table via "*pmid*".<br>Linked to "*hralit_funding*" table via "*funding_id*".<br>Linked to "*hralit_funder_cleaned*" table via "*soa_funder_id*".		
	Notes	This table serves as a junction table to establish many-to-many relationships among publications, fundings, and funders.<br>This table includes uncleaned funder data from PubMed and cleaned funder data from SemOpenAlex.		
hralit_digital_objects	Purpose	To store information about various digital objects related to HRA.		
	Fields	Column	Data type	Description
		do_type	varchar	Type of digital object (e.g., asct-b, omap, 2d-ftu, vascular-geometry, ref-organ).
		do_name	varchar	Name of the digital object.
		do_version	varchar	Version of the digital object.
		do_title	varchar	Title associated with the digital object.
		do_license	varchar	License under which the digital object is released.
		do_publisher	varchar	Entity that published the digital object.
		hubmap_id	varchar	Unique identifier within the HuBMAP (Human BioMolecular Atlas Program).
		do_doi	varchar	Digital Object Identifier for the object.
	Relationships	Linked to "*hralit_creator*" table via "*hubmap_id*".<br>Linked to "*hralit_reviewer*" table via "*hubmap_id*".		
	Notes	The data for this HRA 5th release table is available at https://hubmapconsortium.github.io/ccf-releases/v1.4/docs.		
hralit_asctb_publication	Purpose	To store information about general references and specific references in ASCT+B Tables.		
	Fields	Column	Data type	Description
		organ	varchar	Organ related to the entity being discussed in the publication.
		id	varchar	Identifier for the publication.
		doi	varchar	Digital Object Identifier for the publication.
		notes	varchar	Additional notes or comments related to the publication.
		type	varchar	Type of the publication (e.g., general_publication, reference).
	Relationships	Linked to "*hralit_publication*" table via "*doi*" field.<br>Linked to "*hralit_organ*" table via "*organ*" field.		
	Notes	The data for this table is sourced from the 5th release of the Anatomical Structures, Cell Types, and Biomarker (ASCT+B) Tables.		
hralit_other_publication	Purpose	To store information of publications associated with CZ CELLxGENE and CellMarker.		
	Fields	Column	Data type	Description
		pmid	varchar	Unique identifier for each publication based on the PubMed ID.
		doi	varchar	Digital Object Identifier for the publication.
		source	varchar	Source from which the publication information was obtained (i.e., cxg, cellmarker).

	Relationships	Linked to "*hralit_publication*" table via "*pmid*" field.		
	Notes	The data for this table is sourced from CZ CELLxGene API and CellMarker. CellMarker data is available at http://xteam.xbio.top/CellMarker/download/Human_cell_markers.txt		
hralit_creator	Purpose	To store information about the creators of various digital objects for HRA.		
	Fields	Column	Data type	Description
		organ	varchar	Organ related to the entity being discussed in the publication.
		orcid	varchar	Unique identifier for each creator, based on the ORCID ID system.
		full_name	varchar	Full name of the creator.
		first_name	varchar	First name of the creator.
		last_name	varchar	Last name of the creator.
		do_version	varchar	Version of digital objects related to the creator's contribution.
		do_name	varchar	Name of digital objects related to the creator's contribution.
		do_type	varchar	Type of digital objects related to the creator's contribution.
		hubmap_id	varchar	Unique identifier within the HuBMAP (Human BioMolecular Atlas Program).
	Relationships	Linked to "*hralit_digital_objects* " table via "*hubmap_id*" field.		
	Notes	This table serves as a junction table to establish many-to-many relationships among creators, authors, and digital objects.<br>The data for this table is available at https://hubmapconsortium.github.io/ccf-releases/v1.4/docs.		
hralit_reviewer	Purpose	To store information about the reviewers of various digital objects for HRA.		
	Fields	Column	Data type	Description
		orcid	varchar	Unique identifier for each reviewer, based on the ORCID ID system.
		full_name	varchar	Full name of the reviewer.
		first_name	varchar	First name of the reviewer.
		last_name	varchar	Last name of the reviewer.
		do_version	varchar	Version of digital objects related to the reviewer's contribution.
		do_name	varchar	Name of digital objects related to the reviewer's contribution.
		do_type	varchar	Type of digital objects related to the reviewer's contribution.
		organ	varchar	Organ related to the digital object that the creator created.
		hubmap_id	varchar	Unique identifier within the HuBMAP (Human BioMolecular Atlas Program).
	Relationships	Linked to "*hralit_digital_object_5th_release*" table via "*hubmap_id*" field.		
	Notes	This table serves as a junction table to establish many-to-many relationships among reviewers, authors, digital objects.<br>The data for this table is available at https://hubmapconsortium.github.io/ccf-releases/v1.4/docs.		
hralit_anatomical_structures	Purpose	To store information about different anatomical structures.		
	Fields	Column	Data type	Description
		iri	varchar	An Internationalized Resource Identifier for each anatomical structure, used to reference a specific entity, such as "http://purl.obolibrary.org/obo/UBERON_0006082".
		pref_label	varchar	Preferred label for the anatomical structure.
		type	varchar	The type of the anatomical structure.
	Relationships	Linked to "*hralit_asctb_linkage*" table via "*iri*" field.<br>Linked to "*hralit_triple*" table via "*iri*" field.		
	Notes	The data for this table is sourced from the 5th release of the Anatomical Structures, Cell		

Table	Section	Column	Data type	Description
		Types, and Biomarker (ASCT+B) Tables.		
hralit_cell_types	Purpose	To store information about different cell types.		
	Fields	Column	Data type	Description
		iri	varchar	An Internationalized Resource Identifier for each cell type, used to reference a specific entity, such as "http://purl.obolibrary.org/obo/CL_0000071".
		pref_label	varchar	Preferred label for the cell type.
		type	varchar	The type of the cell type.
	Relationships	Linked to "*hralit_asctb_linkage*" table via "*iri*" field.		
		Linked to "*hralit_triple*" table via "*iri*" field.		
	Notes	The data for this table is sourced from the 5th release of the Anatomical Structures, Cell Types, and Biomarker (ASCT+B) Tables.		
hralit_biomarkers	Purpose	To store information about various biomarkers.		
	Fields	Column	Data type	Description
		iri	varchar	An Internationalized Resource Identifier for each biomarker, used to reference a specific entity, such as "http://identifiers.org/hgnc/6066".
		pref_label	varchar	Preferred label for the biomarker.
		type	varchar	The type of the biomarker.
	Relationships	Linked to "*hralit_asctb_linkage*" table via "*iri*" field.		
		Linked to "*hralit_triple*" table via "*iri*" field.		
	Notes	The data for this table is sourced from the 5th release of the Anatomical Structures, Cell Types, and Biomarker (ASCT+B) Tables.		
hralit_triple	Purpose	To store ontology-related data, potentially including relationships between various biomedical entities.		
	Fields	Column	Data type	Description
		row_id	varchar	Unique identifier for each row, typically in the format of a prefixed namespace and numerical code (e.g., "blood_11", etc.).
		iri	varchar	An Internationalized Resource Identifier for each biomarker, used to reference a specific entity, such as "http://identifiers.org/hgnc/6066".
		pref_label	varchar	Preferred label for the biomarker.
		type	varchar	The type of the biomarker.
		organ	varchar	Organ associated with the entity.
	Relationships	Linked to "*hralit_anatomical_structures*" table via "*iri*".		
		Linked to "*hralit_cell_types*" table via "*iri*".		
		Linked to "*hralit_biomarkers*" table via "*iri*".		
		Linked to "*hralit_organ*" table via "*organ*".		
	Notes	This table serves as a junction table to establish many-to-many relationships among anatomical_structure entities, cell type entities, and biomarker entities.		
		The data for this table is sourced from the 5th release of the Anatomical Structures, Cell Types, and Biomarker (ASCT+B) Tables.		
hralit_donor	Purpose	To store comprehensive information about donors, including both biometric data and medical history.		
	Fields	Column	Data type	Description
		donor_id	varchar	Unique identifier for each donor.
		sex	varchar	Biological sex of the donor.
		age	varchar	Age of the donor.
		death_event	varchar	Event or circumstance leading to the donor's death.
		source	varchar	Source from which the donor information was obtained (i.e., hubmap, cxg, gtex).
		age_unit	varchar	Unit in which the age is measured (e.g., years).

		weight	varchar	Weight of the donor.
		weight_unit	varchar	Unit in which the weight is measured (e.g., kg, pounds).
		height	varchar	Height of the donor.
		height_unit	varchar	Unit in which the height is measured (e.g., cm, inches).
		race	varchar	Race of the donor.
		body_mass_index	varchar	Body Mass Index (BMI) of the donor.
		body_mass_index_unit	varchar	Unit in which the BMI is measured.
		blood_type	varchar	Blood type of the donor (e.g., A, B, AB, O).
		rh_blood_group	varchar	Rhesus (Rh) blood group of the donor (e.g., Rh negative).
		rh_factor	varchar	Rhesus (Rh) factor (e.g., positive or negative).
		kidney_donor_profile_index	varchar	Kidney Donor Profile Index (KDPI) of the donor.
		kidney_donor_profile_index_unit	varchar	Unit in which the KDPI is measured.
		cause_of_death	varchar	Cause of the donor's death.
		medical_history	varchar	Medical history of the donor.
		mechanism_of_injury	varchar	Mechanism of any injury sustained by the donor.
		social_history	varchar	Social history of the donor.
		sex_ontology	varchar	Ontological classification of sex.
		race_ontology	varchar	Ontological classification of race.
	Relationships	Linked to "*hralit_datasets*" table via "*donor_id*".		
	Notes	The data for this table is sourced from the Human BioMolecular Atlas Program (HuBMAP) portal, CZ CELLxGENE portal, and Genotype-Tissue Expression (GTEx) portal.		
hralit_dataset	Purpose	To store detailed information about datasets, including associated donor and sample details, metadata, and dataset status.		
	Fields	Column	Data type	Description
		dataset_id	varchar	Unique identifier for each dataset.
		organ_gtex_id	varchar	GTEx identifier for the organ related to the dataset.
		donor_id	varchar	Identifier for the donor associated with the dataset.
		individual_id	varchar	Identifier for the individual from whom the sample was taken.
		protocols_used	varchar	Protocols used in the generation or analysis of the dataset.
		rin_score_from_paxgene	varchar	RIN score obtained from the PAXgene process.
		rin_score_from_frozen	varchar	RIN score obtained from the frozen sample.
		organ	varchar	Organ from which the sample was taken.
		autolysis_score	varchar	Score indicating the level of tissue autolysis.
		sample_ischemic_time	varchar	Time for which the sample underwent ischemia.
		sample_type	varchar	Type of sample (e.g., normal).
		pathology_notes	varchar	Notes related to pathology findings.
		source	varchar	Source from which the dataset was obtained (i.e., hubmap, cxg, gtex).
		dataset_hubmap_id	varchar	HuBMAP identifier for the dataset.
		dataset_status	varchar	Current status of the dataset (e.g., published).
		dataset_date_time_created	varchar	Date and time the dataset was created.
		dataset_date_tim	varchar	Date and time the dataset was last modified.

e_modified		
dataset_data_types	varchar	Types of data included in the dataset.
dataset_portal_url	varchar	URL to access the dataset in a portal.
first_sample_hubmap_id	varchar	HubMAP identifier for the first sample.
first_sample_submission_id	varchar	Submission identifier for the first sample.
first_sample_uuid	varchar	UUID for the first sample.
first_sample_type	varchar	Type of the first sample.
first_sample_portal_url	varchar	Portal URL for the first sample.
organ_hubmap_id	varchar	HuBMAP identifier for the organ.
organ_submission_id	varchar	Submission identifier for the organ.
organ_uuid	varchar	UUID for the organ.
donor_submission_id	varchar	Submission identifier for the donor.
donor_uuid	varchar	UUID for the donor.
donor_group_name	varchar	Name of the donor group.
rui_location_hubmap_id	varchar	HuBMAP identifier for the RUI location.
rui_location_submission_id	varchar	Submission identifier for the RUI location.
rui_location_uuid	varchar	UUID for the RUI location.
sample_metadata_hubmap_id	varchar	HuBMAP identifier for sample metadata.
sample_metadata_submission_id	varchar	Submission identifier for sample metadata.
sample_metadata_uuid	varchar	UUID for sample metadata.
processed_dataset_uuid	varchar	UUID for the processed dataset.
processed_dataset_hubmap_id	varchar	HuBMAP identifier for the processed dataset.
processed_dataset_status	varchar	Status of the processed dataset.
processed_dataset_portal_url	varchar	Portal URL for the processed dataset.
previous_version_hubmap_ids	varchar	HuBMAP identifiers for previous versions of the dataset.
cxg_dataset_id	varchar	Identifier for the CxG dataset.
dataset_title	varchar	Title of the dataset.
dataset_h5ad_path	varchar	Path to the H5AD file for the dataset.
dataset_total_cell_count	varchar	Total cell count in the dataset.
collection_id	varchar	Identifier for the collection to which the dataset belongs.
collection_name	varchar	Name of the collection.
publication_doi	varchar	DOI for publications related to the dataset.
organ_ontology	varchar	Ontological classification for the organ.
anatomical_structure	varchar	Description of the anatomical structure from which the sample was taken.
anatomical_struct	varchar	Ontological classification for the anatomical structure.

		ure_ontology		
		suspension_type	varchar	Type of suspension used in the sample.
	Relationships	Linked to "*hralit_donor*" table via "*donor_id*".		
		Linked to "*hralit_organ*" table via "*organ*".		
		Linked to "*hralit_publication*" table via "*publication_doi*".		
	Notes	The data for this table is sourced from the Human BioMolecular Atlas Program (HuBMAP) portal, CZ CELLxGENE portal, and Genotype-Tissue Expression (GTEx) portal.		
		This table serves as a junction table to establish many-to-many relationships among datasets, donors, publications.		
hralit_publication_subject	Purpose	To store information about the organ subjects that are the focus of various publications.		
	Fields	Column	Data type	Description
		pmid	varchar	Unique identifier for each publication based on the PubMed ID.
		organ	varchar	The subject of the publication.
	Relationships	Linked to the "*hralit_publication*" table via "*pmid*" field.		
		Linked to the "*hralit_organ*" table via "*organ*" field.		
	Notes	The organ subject is tagged based on the titles and keywords or MeSH terms of the publication.		
hralit_publication_author	Purpose	To establish relationships between publications and their respective authors		
	Fields	Column	Data type	Description
		pmid	varchar	Unique identifier for each publication based on the PubMed ID.
		author_id	varchar	Unique identifier for each author.
	Relationships	Linked to "*hralit_publication*" table via "*pmid*".		
		Linked to "*hralit_author*" table via "*author_id*".		
	Notes	This table serves ƒas a junction table to establish many-to-many relationships between publications and authors.		
hralit_organ	Purpose	To store information about organs in 5th release ASCT+B Tables.		
	Fields	Column	Data type	Description
		organ	varchar	Organ name.
	Relationships	Linked to "*hralit_triple*" table via "*organ*".		
		Linked to "*hralit_publication_subject*" table via "*organ*".		
	Notes	The data for this table is available at https://hubmapconsortium.github.io/ccf-releases/v1.4/docs.		
hralit_asctb_linkage	Purpose	To store ontology-related data, potentially including relationships between various biomedical entities.		
	Fields	Column	Data type	Description
		s_iri	varchar	IRI for the source.
		relationship	varchar	Linkage between source and target.
		t_iri	varchar	IRI for the target.
	Relationships	Linked to "*hralit_anatomical_structures*" table via "*s_iri*".		
		Linked to "*hralit_cell_types*" table via "*s_iri*".		
		Linked to "*hralit_biomarkers*" table via "*s_iri*".		
		Linked to "*hralit_anatomical_structures*" table via "*t_iri*".		
		Linked to "*hralit_cell_types*" table via "*t_iri*".		
		Linked to "*hralit_biomarkers*" table via "*t_iri*".		
	Notes	This table serves as a junction table to establish many-to-many relationships among anatomical_structure entities, cell type entities, and biomarker entities.		
		The data for this table is sourced from the 5th release of the Anatomical Structures, Cell Types, and Biomarker (ASCT+B) Tables.		

Table S2. Record count statistics for 22 HRAlit database tables.

Table	Records	Columns
hralit_anatomical_structures	4,378	3
hralit_asctb_publication	1,288	5
hralit_asctb_linkage	25,277	3
hralit_author	583,117	5
hralit_author_institution	464,043	2
hralit_biomarkers	2,522	3
hralit_cell_types	1,395	3
hralit_creator	550	9
hralit_dataset	7,337	46
hralit_digital_objects	295	8
hralit_donor	4,639	24
hralit_funder_cleaned	6,427	3
hralit_funding	917,061	2
hralit_institution	26,235	5
hralit_organ	31	1
hralit_other_publication	1,823	3
hralit_pub_funding_funder	2,632,888	6
hralit_publication	7,103,180	5
hralit_publication_author	1,079,698	2
hralit_publication_subject	7,898,258	2
hralit_reviewer	602	9
hralit_triple	178,893	4
**Total**	**20,939,937**	**153**

Note that hralit_asctb_linkage, hralit_author_institution, hralit_pub_funding_funder, hralit_publication_author, hralit_publication_subject, hralit_triple are the 6 junction tables.

Table S3. Node count statistics for HRAlit database tables.

Name	#Nodes
anatomical structure	4,279
author	583,117
biomarker	2,089
CellMarker/CxG/GTEx publication*	1,816
cell type	1,210
creator	101
dataset	1,816
digital object	295
donor	4,639
funded project	896,680
funder (cleaned)	6,427
funder (uncleaned)	63,691
institution (cleaned)	26,235
reviewer	99
organ	31
publication	7,103,180
publication in ASCT+B Tables	1,057
**Total**	**8,696,762**

Note that in CellMarker, CxG, and GTEx publications, the number refers to the publications with DOIs.

Table S4. Linkage count statistics for HRAlit database for 22 relationship types.

Relationships	#Linkages
anatomical structure & anatomical structure	4,795
author & institution	464,043
biomarker & cell typeslee	5,753
CellMarker/CxG/GTEx publication & publication	1,817
cell type & anatomical structure	13,471
cell type & cell type	1,258
creator & digital object	550
dataset & donor	7,337
dataset & publication	5,228
funding & cleaned funder	147,202
funding & uncleaned funder	888,249
publication & author	1,079,698
publication & funding	2,616,446
publication & organ	7,898,258
publication in ASCT+B Tables & publication	472
publication in ASCT+B Tables & organ	1,288
reviewer & digital object	602
triple (anatomical structure & cell type & biomarker)	17,092
triple & organ	17,092
**Total**	**13,170,651**
